# Fate of stable hips after prophylactic femoral varization osteotomy in patients with cerebral palsy

**DOI:** 10.1186/s12891-018-2049-z

**Published:** 2018-04-27

**Authors:** Ki Hyuk Sung, Soon-Sun Kwon, Chin Youb Chung, Kyoung Min Lee, Jaeyoung Kim, Seung Yeol Lee, Moon Seok Park

**Affiliations:** 1 Department of Orthopaedic Surgery, Seoul National University Bundang Hospital, 82 Gumi-ro 173 Beon-gil, Bundang-Gu, Sungnam, Gyeonggi 13620 Korea; 2 Department of Mathematics, College of Natural Sciences, Ajou University, Suwon, Gyeonggi Korea; 3 Department of Orthopaedic Surgery, H-Plus Yangji Hospital, Seoul, Korea; 4 Department of Orthopaedic Surgery, Ehwa Womans Mokdong Hospital, Seoul, Korea

**Keywords:** Prophylactic femoral varization osteotomy, Stable hip, Displaced hip, Cerebral palsy, Hip reconstructive surgery

## Abstract

**Background:**

Concurrent prophylactic femoral varization osteotomy (FVO) for stable hips has been performed in patients with cerebral palsy (CP) undergoing hip reconstructive surgery for the contralateral displaced hip. However, there is currently a lack of studies investigating the outcome after the prophylactic FVO in stable hip. This study investigated the outcomes after FVO in stable hips with CP and influencing factors. In addition, this study compared the outcomes with those after hip reconstructive surgery in the contralateral displaced hip.

**Methods:**

This study included 119 CP patients with 224 hips (80 stable, 144 displaced) undergoing hip reconstructive surgery including FVO. Migration percentage (MP), neck-shaft angle (NSA), and head-shaft angle (HSA) were measured through preoperative and follow-up hip radiographs. All hips were divided into the stable (MP ≤ 33%) and displaced hip groups (MP > 33%) according to the preoperative radiographs, and the annual changes in the radiographic indices after FVO were analyzed.

**Results:**

In stable hip group, MP did not significantly increase over time (*p* = 0.057) after prophylactic FVO. In displaced hip group, MP significantly increased over time (1.6%/year, *p* < 0.001). MP was significantly decreased in cases of concomitant Dega pelvic osteotomy in both stable (14.5%, *p* < 0.001) and displaced hips (18.9%, *p* < 0.001).

**Conclusions:**

Prophylactic FVO in the stable hip in patients with CP showed good surgical outcomes, without a risk of hip displacement throughout the follow-up duration, while hip reconstructive surgery in the displaced hip was associated with a risk of increased hip displacement.

## Background

The prevalence of hip displacement (subluxation or dislocation) in patients with cerebral palsy (CP) ranges from 1 to 79% according to the severity of involvement [1]. Hip displacement can lead to pain, difficulties in performing perineal hygiene, pressure ulcers, lower limb fractures, and loss of balance to sit [1, 2]. When the subluxation is severe or dislocation is present, hip reconstructive surgery consisting of femoral varization osteotomy (FVO), with or without pelvic osteotomy, is indicated [3, 4].

In cases of unilateral hip displacement, there is some controversy regarding the appropriate treatment for the contralateral stable hip. Some investigators have advocated hip reconstructive surgery of the involved hip only [3, 5]. However, other authors suggest bilateral surgery because of the increased risk of progressive migration of the contralateral hip [6–9]. Canavese et al. reported that 44% of severely involved patients with CP undergoing unilateral FVO required subsequent bony surgical management of the contralateral hip for subluxation or dislocation before reaching skeletal maturity [8]. Our recent study using a decision analysis model demonstrated that concurrent prophylactic FVO for the contralateral stable hip in individuals with CP undergoing hip reconstructive surgery was better than closed observation from a medical perspective [7]. Therefore, our institution has been performing concurrent prophylactic FVO for stable hips in patients with CP who had undergoing hip reconstructive surgery for the contralateral displaced hip.

A number of studies have investigated the outcome after hip reconstructive surgery in cases of hip instability in patients with CP and showed good surgical outcomes [10–14]. However, several studies have also reported recurrence of hip displacement after reconstructive surgery and that the preoperative degree of hip displacement, functional level of the patient and, uncorrected acetabular dysplasia were factors associated with the postoperative outcomes [15–17]. Nevertheless, there is currently a lack of studies investigating the outcome after the prophylactic FVO in stable hip.

Therefore, we performed this study to investigate the outcomes after prophylactic FVO in stable hips in patients with CP in terms of the radiographic parameters and influencing factors. In addition, we compared the outcomes with those after hip reconstructive surgery in the contralateral displaced hip.

## Methods

### Participants

The inclusion criteria were as follows: (1) CP patients with hip displacement who underwent hip reconstructive surgery including FVO from May 2003 to February 2015, (2) patients with a minimum of 1 year of follow-up, and (3) patients with availability of preoperative hip radiographs and radiographs obtained during at least two follow-up evaluations. The exclusion criteria were as follows: (1) previous hip surgery that resulted in a change in the natural shape of the hip and (2) inadequate preoperative or postoperative radiographs available for measurement.

Data regarding the age at surgery, sex, duration of follow-up, anatomical type of CP (diplegia vs. quadriplegia), Gross Motor Function Classification System (GMFCS) level, and whether a concomitant Dega pelvic osteotomy was performed were obtained from the patients’ medical records.

### Operative procedures

At our institution, hip reconstructive surgery including FVO is performed in displaced hips with a migration percentage (MP) of > 33%. All patients underwent medial soft tissue release of the adductor longus tendon; if the abduction angle obtained was not > 30°, additional soft-tissue release, including of the adductor brevis, gracilis, and pectineus, was performed [16]. For the contralateral stable hip (MP ≤ 33%), prophylactic FVO was routinely performed. All patients underwent FVO and the osteotomy site was internally fixated with a pediatric locking compression plate (Synthes, Zuchwil, Switzerland) or a blade plate (Stryker, Selzach, Switzerland) according to the surgeon’s preference. After FVO, if concentric reduction was not achieved on intraoperative fluoroscopic examination, open reduction of the hip joint including capsulorrhaphy, removal of the ligamentum teres and pulvinar, and resection of the transverse acetabular ligament was additionally performed. In cases of radiographic findings of acetabular defects preoperatively, a modified Dega pelvic osteotomy was performed [18, 19]. Postoperatively, a short leg cast and abduction bar were applied to maintain hip abduction position for 4–6 weeks. Hardware removal was performed more than 6 months after the initial operation.

### Consensus building and radiographic indices

A consensus building session to select and define the radiographic indices was held by six orthopedic surgeons (MSP, KML, KHS, JYK, BCJ, and SJM), with orthopedic experiences of 16, 14, 12, 7, 5, and 4 years, respectively. Previous studies were reviewed [1, 16, 20–22], and 5 parameters that were relevant to measuring hip displacement were extracted, namely the neck-shaft angle (NSA), head-shaft angle (HSA), MP, acetabular index, and center-edge angle on hip radiographs. Of these, the acetabular index and center-edge angle are known to be unable to predict hip displacement [1, 21]; therefore, the remaining three parameters, NSA, HSA, and MP, were finally selected as the relevant radiographic measurements.

Hip radiographs were obtained from each patient in the supine position and with the hips rotated internally by approximately 30°. Radiographs were taken using a UT 2000 unit (Philips, Eindhoven, the Netherlands) under the following conditions: source-to-image distance of approximately 100 cm, 60 kVp, and 10 mAs.

**Fig. 1:**
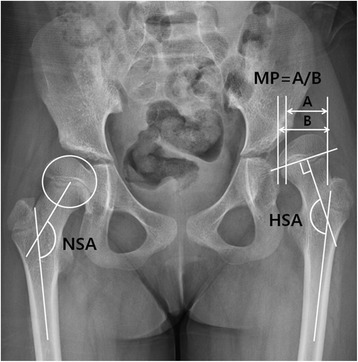
Hip internal rotation view. For the right hip, neck-shaft angle (NSA) was defined as the angle between a line passing through the center of the femoral shaft and another line connecting the femoral head center and the midpoint of the femoral neck. The femoral head center was the center of the largest best-fitting circle inside the femoral head. For the left hip, migration percentage was calculated by dividing the width of the femoral head lateral to Perkin’s line (A) by the total width of the femoral head (B). Head-shaft angle (HSA) was defined as the angle between a line passing through the center of the femoral shaft and another line perpendicular to the proximal femoral physis passing through the center of the proximal femoral epiphysis

The NSA of the femur was measured as the angle between a line through the midpoint of the femoral shaft and another line through the femoral head center and midpoint of the femoral neck, on anteroposterior hip internal rotation radiographs. The femoral head center was defined as the center of the best fitting outer circle into the femoral head. The HSA was formed by a line drawn through the femoral shaft midway and another line perpendicular to the proximal femoral physis passing through the center of the proximal femoral epiphysis. The MP was defined as the ratio where the amount of the femoral head lateral to the Perkins line was divided by the total femoral head. When the lateral margin of the femoral head was medial to Perkins’ line and the MP is in fact a negative value, it was given a value of 0%. When the whole femoral head was lateral to Perkins’ line, the MP was considered as 100% (Fig. 1) [16, 20, 23, 24].

### Reliability testing and radiographic measurements

After consensus building, reliability testing was performed prior to the main measurements. Three orthopedic surgeons (JYK, SGM, AZ), with 7, 4, and 4 years of orthopedic experience, respectively, assessed the interobserver reliability of the measurements of the radiographic indices. A prior sample size estimation by precision analysis indicated that a minimum of 36 hip radiographs needed to be assessed. The three examiners measured the radiographic indices independently, without knowledge of the patients’ clinical information and the other orthopedic surgeons’ measurements. Four weeks after the measurements, one surgeon (JYK) repeated the radiographic measurements to assess the interobserver reliability. All measurements were collected by a research assistant, who did not participate in the study.

After reliability testing, one of the authors (JYK) measured all preoperative and periodic follow-up hip radiographs. All measurements were taken using a picture archiving and communication system (Impax; Agfa, Antwerp, Belgium). The hips were divided into the stable and displaced hip groups according to the preoperative hip radiograph findings. The stable and displaced hip groups were defined as hips with a preoperative MP ≤33% and > 33%, respectively. The annual changes in the radiographic indices after FVO were analyzed by using linear mixed model (LMM) analysis.

### Building the linear mixed model

LMMs are parametric linear models for longitudinal data that quantify the relationships between a continuous dependent variable and various predictor variables, thereby providing a simple and effective way to incorporate within-subject and between-subject variations and the correlation structure of longitudinal data [25]. LMMs consist of both fixed effects and random effects. The fixed effects represent categorical levels that are measurable and are not random, such as sex. Random effects are factors that can be specified for individuals within a population and that account for the variation within individuals. Therefore, it can be expected that, by using an LMM application, estimation of the annual changes in the radiographic hip indices may confer more practical information to clinicians [26].

In this study, the NSA, HAS, and MP were first adjusted for multiple factors with use of an LMM, with sex, age at surgery, GMFCS level, anatomical type of CP, and concomitant Dega pelvic osteotomy as the fixed effects, and follow-up duration, laterality (left or right), and each subject as the random effects. The estimation method used restricted maximum likelihood estimations to produce unbiased estimators. Next, an LMM was developed to estimate the radiographic changes by incorporating the linear follow-up duration effect, sex, age at surgery, GMFCS level, anatomical type of CP, concomitant Dega pelvic osteotomy, and side of the hip operation as covariates. By examining the individual pattern of the radiographic changes along with the follow-up time, a model with a random slope and a random intercept was suggested. Subsequently, the linear effects of follow-up duration, sex, age at the time of the operation, and laterality were integrated to evaluate the estimates of the three measurements. The models were compared by using the Akaike information criterion and the Bayesian information criterion. Consequently, the models with the covariate effects of follow-up duration, age at surgery, sex, GMFCS level, anatomical type, concomitant Dega osteotomy, and laterality were accepted as valid for estimation of these measurements.

### Statistical methods

A sample size analysis was carried out to determine the minimum number of patients required for reliability testing. The reliability was calculated with the use of intraclass correlation coefficients (ICCs) at a target value of 0.8. The 95% confidence interval (CI) was set to 0.2, and, using Bonett’s approximation, the minimum sample size was set as 36 hips [27].

The ICCs and their 95% CIs were used to summarize the interobserver and intraobserver reliabilities of NSA, HAS, and MP and were calculated in the setting of a two-way random-effects model, assuming a single measurement and absolute agreement [28, 29]. An ICC value of 1 indicates perfect reliability and an ICC of > 0.8 indicates excellent reliability. Descriptive statistics, such as the mean and standard deviation, were used to summarize the patient demographics and radiographic measurements. To consider bilateral cases, an LMM was applied for statistical analysis. [30] All statistical analyses were conducted using SAS 9.4.2 (SAS Institute, Cary, NC, USA); all statistics were two-tailed, and *p*-values < 0.05 were considered significant.

## Results

A total of 119 patients (224 hips) were finally included in this study, and 1569 radiographs were evaluated. Twenty-five patients had bilateral hip displacement and 94 patients had unilateral hip displacement. Among the 94 stable hips, prophylactic FVO was performed in 80 hips because the parents of 14 patients refused to the prophylactic FVO. Hip reconstructive surgery for the displaced hip was performed in 144 hips. The majority of patients showed quadriplegia (101 patients) based on the anatomical classification, and GMFCS level V (56 patients) based on the functional classification. The mean age at surgery was 8.9 ± 2.7 years and the mean follow-up duration was 3.3 ± 2.7 years. The mean number of follow-up examinations per patients was 6 (range, 2–15). Eighteen patients (15.1%) had a history of selective dorsal rhizotomy and 94 hips (42.0%) underwent concomitant Dega pelvic osteotomy (Table 1). The complications after surgery included supracondylar fracture of the distal femur in 1 patient and subtrochanteric fracture after implant removal in 1 patient. In addition, 5 hips (2.23%) were re-dislocated (1 in stable hip and 4 in displaced hip), of which, 4 were re-operated.

**Table 1:** Summary of patient data

Parameters	Values
Male/Female	78 / 41
Anatomical type (diplegia/guadriplegia)	18 / 101
GMFCS level (II-III/I*V*/V)	18 / 45 / 56
Age at surgery (years)	8.9 ± 2.7 (2.8 to 16.5)
Follow-up duration (years)	3.3 ± 2.7 (1 to 11.9)
Laterality (Right / Left)	112 / 112
Unilateral / Bilateral hip displacement	94 / 25
Concomitant Dega osteotomy (Yes / No)	94 / 130

*GMFCS* Gross Motor Function Classification System

Radiographic measurements showed good to excellent inter-observer and intra-observer reliabilities (ICC, 0.729–0.885). The NSA, HSA, and MP were significantly improved after FVO in both groups (all *p* < 0.001). There was a significant difference in the preoperative MP between the stable and displaced hip groups (*p* < 0.001). However, there were no significant differences in the preoperative NSA (*p* = 0.387) and HAS (*p* = 0.695) between the two groups (Table 2).

**Table 2:** Summary of radiographic measurements

Radiographic measurement	Total	Stable hip group (80 hip)	Displaced hip group (144 hip)	*p*-value
Neck-shaft angle (degree)				
Preoperative	151.3 ± 8.5	150.7 ± 7.4	151.7 ± 9.0	0.387
Immediate postoperative	120.5 ± 9.6	124.7 ± 7.4	117.9 ± 9.8	< 0.001
Final follow-up	126.9 ± 14.2	129.0 ± 11.9	125.7 ± 15.2	0.094
Head-shaft angle (degree)				
Preoperative	158.7 ± 10.3	159.1 ± 9.4	158.5 ± 10.8	0.695
Immediate postoperative	130.1 ± 13.3	133.2 ± 10.0	128.4 ± 14.5	0.008
Final follow-up	135.3 ± 16.7	137.1 ± 14.0	134.3 ± 18.0	0.231
Migration percentage (%)				
Preoperative	51.1 ± 28.3	21.8 ± 8.1	67.3 ± 21.7	< 0.001
Immediate postoperative	2.7 ± 6.4	3.6 ± 5.6	2.3 ± 6.7	0.148
Final follow-up	14.0 ± 14.3	15.1 ± 11.2	13.3 ± 15.7.	0.369

**Fig. 2:**
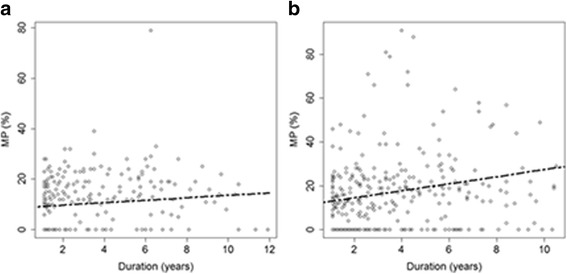
Scatterplots showing the migration percentage (MP) according to follow-up duration for stable hip (**a**) and displaced hip group (**b**). The solid lines represent an estimation of the recovery by a linear follow-up duration effect

In the stable hip group, MP was not significantly increased over time (0.5%/year, *p* = 0.057) after prophylactic FVO (Fig. 2a). Moreover, the MP was not significantly affected by sex, age at surgery, the GMFCS level, and the anatomical type of CP. However, it was significantly decreased in cases of concomitant Dega pelvic osteotomy (14.5%, *p* < 0.001) (Table 3). In the displaced hip group, the MP after hip reconstructive surgery was significantly increased over time (1.6%/year, *p* < 0.001) and was decreased in cases of concomitant Dega pelvic osteotomy (18.9%, *p* < 0.001; Fig. 2b). However, it was not significantly affected by the patients’ sex, age at surgery, GMFCS level, and anatomical type of CP (Table 4).

**Table 3:** Factors affecting migration percentage after prophylactic femoral varization osteotomy in stable hip

	Estimate (%)	95% CI	SE	P-value
Intercept	8.7	−0.0 to 17.5	4.5	
Follow-up duration (year)	0.5	−0.0 to 1.0	0.3	0.057
Age at surgery	−0.4	−0.9 to 0.2	0.3	0.218
Sex	1.2	−2.0 to 4.5	1.7	0.459
GMFCS level III-IV	−2.0	−6.8 to 2.8	2.4	0.405
GMFCS level III-V	−2.8	−8.1 to 2.5	2.7	0.308
Anatomical type	2.2	−2.1 to 6.5	2.2	0.322
Laterality	−2.3	−4.9 to 0.2	1.3	0.073
Concomitant Dega osteotomy	14.5	10.6 to 18.5	2.0	< 0.001

A linear mixed model was used to estimate factors affecting migration percentage

*CI* confidence interval, *SE* standard error, *GMFCS* Gross Motor Function Classification System

**Table 4:** Factors affecting migration percentage after femoral varization osteotomy in displaced hip

	Estimate (%)	95% CI	SE	P-value
Intercept	11.4	1.2 to 21.6	5.2	
Follow-up duration (year)	1.6	1.0 to 2.2	0.3	< 0.001
Age at surgery	−0.4	−1.0 to 0.2	0.3	0.194
Sex	−3.3	−7.0 to 0.4	1.9	0.082
GMFCS level III-IV	−3.9	−9.1 to 1.3	2.7	0.139
GMFCS level III-V	0.0	−5.1 to 5.1	2.6	0.998
Anatomical type	−3.6	−9.4 to 2.2	2.9	0.222
Laterality	−3.8	−6.7 to −1.0	1.5	0.009
Concomitant Dega osteotomy	18.9	14.8 to 23.1	2.1	< 0.001

A linear mixed model was used to estimate factors affecting migration percentage

*CI* confidence interval, *SE* standard error, *GMFCS* Gross Motor Function Classification System

HSA was not significantly increased over time in the stable hip group (*p* = 0.451), but was significantly increased in the displaced hip group (0.8°/year, *p* = 0.039). NSA was significantly increased over time in both the stable (0.9°/year, *p* = 0.005) and displaced hip groups (1.9°/year, *p* < 0.001).

## Discussion

To our knowledge, this investigation is the first to evaluate the outcomes after prophylactic FVO for stable hips in patients with CP. The present study demonstrated that there was no annual increase in the MP after prophylactic FVO in stable hips with CP, whereas there was a significant increase after FVO in displaced hips.

There were some limitations for this study. First, radiographic parameter, MP, which we used for evaluating surgical outcome, is not exact representation of the degree of pain or function. However, MP has been known to be the most objective, accepted, and reproducible measurement of hip displacement, and is little influenced by the rotational position of the femur [9, 31]. In addition, high MP has been known to be related with hip pain [17, 32]. Seconds, other variable such as scoliosis may affect the outcome after FVO. However, data regarding scoliosis were not available because of the retrospective design of this study. Thirds, this was a retrospective study; therefore, some patients had short follow-ups and the follow-up intervals varied. We used an LMM to overcome the unbalanced structure of our data set and focused on annual changes of the radiographic parameters and the factors that could influence these annual changes. Our results using an LMM suggested a trend of change in measurements on the hip radiographs of the CP patients. We believe that these findings can inform physicians of the prognosis of hip reconstructive surgery in CP patients and that including these patients in our study is reasonable. Fourth, although the *p*-value of the annual change in MP according to the follow-up duration did not reach the significance level of 0.05, it was possibly of marginal significance (*p* = 0.057). Thus, a significant association between MP and follow-up duration is possible owing to the longer follow-up duration in the stable hip group (3.6 years) than in the displaced hip group (3.0 years). However, the annual increase in MP was only 0.5% in the stable hip group after prophylactic FVO, and its clinical impact was minimal.

Some surgeons advocate hip reconstructive surgery for the involved hip only [3, 5], whereas others believe that bilateral surgery including prophylactic FVO in the contralateral stable hip should be performed to prevent progressive migration of the stable hip [6–9]. Unilateral subluxation is often associated with pelvic obliquity, with the affected side of the pelvis being elevated. When this hip is surgically corrected, this may result in an alteration in the balance of the forces that control pelvic orientation wherein the opposite side of the pelvis becomes elevated, thereby placing the contralateral hip at risk for progressive subluxation [8].

Noonan et al. evaluated the fate of non-operated hips in 35 patients who underwent surgical stabilization for unilateral hip displacement. Of these, 26 (74.3%) developed subluxation or dislocation. The authors thus recommended bilateral surgery in patients with young age (< 6 years) or an MP > 20% [6]. Carr et al. found that non-ambulators had an increased risk of deterioration of the non-operated hip following unilateral surgery [33], and Shukla et al. reported contralateral hip subluxation in 28% of cases after unilateral hip reconstruction in children with CP [34]. In addition, they found that predictors of contralateral hip subluxation included a lack of contralateral soft tissue release, reversal of pelvic obliquity, and larger initial contralateral MP (> 25%). Canavese et al. reported that bilateral surgery should be considered in GMFCS IV and V CP patients with unilateral hip displacement, even if the contralateral hip appears normal on radiographic examinations [8]. The present study showed that there was no recurrence of hip displacement after prophylactic FVO. Therefore, we think that concurrent prophylactic FVO in the contralateral stable hip could be considered at the time of hip reconstructive surgery of the displaced hip to prevent progressive displacement of the stable hip.

While a number of studies have shown good surgical outcomes after hip reconstructive surgery for hip displacement in patients with CP, several studies also have reported recurrence of hip displacement after hip reconstructive surgery and investigated the risk factors [10–17]. Khalife reported that insufficient correction of preexisting valgus and uncorrected acetabular dysplasia were the main risk factors for recurrent dislocation after FVO [15]. Rutz et al. reported good surgical outcomes after hip reconstructive surgery in 168 hips with an MP of > 30%. In addition, they found that the only factor affecting the postoperative outcome was the preoperative MP [17]. Dhawale et al. showed improvements in the long-term radiographic outcomes after hip reconstructive surgery in quadriplegic CP patients; however, 45.5% of the hips eventually required revision surgery [13]. Bayusentono et al. investigated the recurrence of hip displacement after reconstructive surgery and the influencing factors in CP patients. The authors found that the MP did not change significantly in patients with GMFCS level II or III, whereas it increased significantly by 2.0% and 3.5% per year in patients with GMFCS levels IV and V, respectively. Therefore, they recommended periodic monitoring and follow-up for recurrence of hip displacement in patients with GMFCS levels IV and V [16].

On the other hands, there is a lack of studies investigating the outcomes after prophylactic FVO, although a number of surgeons have recommended concomitant prophylactic FVO for the contralateral stable hip during hip reconstructive surgery for a displaced hip. Oh et al. evaluated the long-term outcomes after FVO in 61 hips, including 24 non-subluxated hips treated by FVO as a balancing procedure. They found satisfactory outcomes in all non-subluxated hips and reported that the preoperative MP significantly influenced the unsatisfactory outcomes in the displaced hips. In addition, they recommended FVO with pelvic osteotomy if acetabular dysplasia was present [2]. In the current study, prophylactic FVO for stable hips resulted in good outcomes in terms of the radiographic indices. In addition, there was no significant increase in the annual MP during the follow-up duration, contrary to the findings in the displaced hips.

Further, our findings showed that concomitant Dega osteotomy in both the stable and displaced hips was significantly associated with the surgical outcomes in terms of the MP. In our institute, we routinely perform hip computed tomography for preoperative evaluation of hip dysplasia. Based on the findings of this examination, concomitant Dega osteotomy was performed in 10 stable and 84 displaced hips. Among the 10 stable hips, 7 and 3 hips were found in patients with GMFCS levels V and IV, respectively. While a positive relationship between acetabular dysplasia and MP has been reported [35], there is, to our knowledge, no previous study on the relationship between the functional level of patients and acetabular dysplasia; therefore, further studies regarding this issue are required.

## Conclusion

Prophylactic FVO in the stable hip in patients with CP showed good surgical outcomes, without a risk of hip displacement throughout the follow-up duration, while hip reconstructive surgery in the displaced hip was associated with a risk of increased hip displacement.

## Abbreviations

CP: Cerebral palsy
FVO: Femoral varization osteotomy
GMFCS: Gross Motor Function Classification System
HSA: Head-shaft angle
LMM: Linear mixed model
MP: Migration percentage
NSA: Neck-shaft angle
